# Augmented reality as multimedia: the case for situated vocabulary learning

**DOI:** 10.1186/s41039-016-0028-2

**Published:** 2016-01-20

**Authors:** Marc Ericson C. Santos, Arno in Wolde Lübke, Takafumi Taketomi, Goshiro Yamamoto, Ma. Mercedes T. Rodrigo, Christian Sandor, Hirokazu Kato

**Affiliations:** 1 Interactive Media Design Laboratory, Graduate School of Information Science, Nara Institute of Science and Technology, 8916-5 Takayama, Ikoma, Nara 630-012 Japan; 2 Ateneo Laboratory for the Learning Sciences, Ateneo de Manila University, Katipunan Ave., Quezon City, Metro Manila 1108 Philippines

**Keywords:** Augmented reality, Multimedia learning, Ubiquitous learning, Vocabulary learning

## Abstract

Augmented reality (AR) has the potential to create compelling learning experiences. However, there are few research works exploring the design and evaluation of AR for educational settings. In our research, we treat AR as a type of multimedia that is situated in authentic environments and apply multimedia learning theory as a framework for developing our educational applications. We share our experiences in developing a handheld AR system and one specific use case, namely, situated vocabulary learning. Results of our evaluations show that we are able to create AR applications with good system usability. More importantly, our preliminary evaluations show that AR may lead to better retention of words and improve student attention and satisfaction.

## Introduction

Augmented reality (AR) is the seamless integration of virtual objects and real environments (Azuma 1997). In other words, in AR, computer-generated information is placed in the world as if they co-exist with real objects. It is an emerging technology that is finding applications in education because of its possible benefits to teaching and learning (Wu et al. 2013). However, AR’s practical uses are relatively not well understood compared to those of virtual reality and other technologies (Joseph and Uther 2009). Moreover, few research works have been conducted to substantiate AR’s benefits to learning (Ibanez et al. 2014).

Research works argue that AR’s strengths and therefore its applicability to education are embodied cognition (Yang and Liao 2014; Kaufmann et al. 2000; Kaufmann 2002) and interactivity (Ibanez et al. 2014; Di Serio et al. 2013). As Specht et al. (2011) explained, AR affords new ways of intuitively interacting with information. Another more fundamental advantage of AR that is not explored as much is the manner of displaying visual information. AR is useful for presenting the explicit relationship of virtual contents to objects found in the real world. For example, Matsutomo et al. (2012) use AR for displaying virtual magnetic fields on physical magnets. Another example is the system of Tarng and Ou (2012) for animating the life cycle of a virtual butterfly on a real plant. Aside from embodied interactions with digital information, researchers have shown some evidence that presenting digital information together with the context of a real environment helps memorization (Fujimoto et al. 2013; Fujimoto et al. 2012). They argue that AR has the potential to ease cognitive load and that using AR allows users to form memory retrieval cues based on the real environment.

Dede (2011) explains that AR is useful for supporting ubiquitous learning in authentic environments. Ubiquitous learning usually involves the use of mobile devices, such as smartphones (Joseph and Uther 2009). Based on the location or other context data of the user, the system can provide some learning content. The role of AR in ubiquitous learning is to present the information onto the real environment thereby creating a stronger connection between the digital content and the real environment. Currently, handheld devices like smartphones are already equipped with cameras and other sensors, enough processing power, and large screens for delivering AR learning experiences (Billinghurst and Duenser 2012). For example, Kamarainen et al. (2013) used AR as a feature of their smartphone-based system to support a field trip in a local pond.

As of the time of this writing, though, there has been little empirical evidence collected to substantiate or refute AR’s potential as a usable carrier of educational content. In a review conducted in 2013, Santos et al. (2014a) found only seven research articles reporting evidence of AR’s effectiveness in improving learning outcomes. In this review, the researchers observed that AR’s impact on learning outcomes vary from a small negative effect to a large positive effect. There are many factors attributed to this variation, such as the comparison being made and the appropriate matching of the technology to pedagogical needs. However, even with the current state of AR, researchers already report that AR has positive effects on motivational factors of attention and confidence (Di Serio et al. 2013).

Given that AR is useful for presenting information relevant to places, AR is a good match for teaching culture and languages (Liu 2009; Liu and Tsai 2013). In this research, we limit language learning to vocabulary learning as the target of AR. In our approach, we based the requirements of our system on multimedia learning theory, previous vocabulary learning systems, and teacher’s feedback on AR. AR is a kind of multimedia that is situated in an authentic environment (Santos et al. 2014d). As such, multimedia learning theory (Mayer 2009; Mayer 2005) can be applied for designing and evaluating AR’s benefits to learning. After implementing the system, we conducted system usability evaluations using general usability scales and a usability scale designed for handheld AR. In our investigation, we reiterated some design guidelines for applying AR to education and added our own design goals. Finally, we evaluated student learning outcomes and student motivation with our application.

The goal of this study is threefold: We would like to (1) develop an AR application, (2) test its usability, and (3) test its effects on learning. To these ends, we demonstrate our development and evaluation framework for prototyping AR learning experiences. We apply AR to the task of memorizing vocabulary words and test AR’s effect on both learning and student motivation. Finally, because there is little literature substantiating the benefits of AR to learning, we test AR’s effectiveness as a platform for a memorization task and examine its impact on student motivation.

## Augmented reality for learning

The general public is becoming more familiar with AR mainly because of AR browsers used for conveying a variety of location-based information (Grubert et al. 2011). Currently, people use some AR browsers to see virtual labels and symbols integrated with a live video feed of the real environment. This makes understanding location-related information, such as names of buildings, distances of restaurants, and arrows for navigation, easier (Fujimoto et al. 2012). In the case of situated vocabulary learning, instead of displaying names and directions, we designed a system that displays words and animations to teach new vocabulary words that are relevant to the objects found within the environment.

Several AR systems have also been developed for educational settings (Santos et al. 2014a). One important work is Construct3D (Kaufmann et al. 2000; Kaufmann 2002) which uses AR to teach students mathematics and geometry concepts. AR is suitable for this purpose because students can interact naturally with three-dimensional shapes without the use of a mouse and keyboard. While wearing a head-mounted display, students move around virtual shapes and perform operations on them. Moreover, the students see the same virtual shapes allowing them to work together on the same target. Although Construct3D take advantage of embodied cognition and collaborative learning, these applications do not use AR for displaying the relationship of the virtual contents to the real environment. In our work, we exploit such AR features by teaching vocabulary through the relationship between virtual objects and the real environment.

AR running on handheld devices can be used for displaying content in big environments. Handheld AR has gained attention in the field of educational technology because of its advantages when applied in ubiquitous learning (Dede 2011), situated cognition (Specht et al. 2011), and collaboration (Li et al. 2011). Billinghurst and Duenser (2012) explain that handheld AR technology is already mature for this application. AR software can already run on mobile phones equipped with fast processors, big display screens, data connections, built-in cameras, and other sensors. Billinghurst and Duenser (2012) call for more interdisciplinary research to ground AR applications in learning theories. For our experiments, we designed AR applications for learning Filipino and German words by applying the principles of multimedia learning theory (Mayer 2009) and its related research. Moreover, we considered some feedback from teachers and school administrators in order to make a practical AR application.

## Vocabulary learning systems

Mastering a foreign language relies heavily on building vocabulary necessary for listening, reading, speaking, and writing (Yang 2012). Several creative approaches have been developed to support such vocabulary learning, including hypertext annotations in e-learning (Chen et al. 2013), collaborative multimedia (Joseph et al. 2005), word games (Lin et al. 2008), virtual environments (Pala et al. 2011), and interactions with robots (Wu et al. 2008). The instructional designs for these prototypes leverage on three main strategies, namely, repetition, engagement, and context. Acquiring new words requires repeated exposure to those words (Webb 2007). This includes both memory rehearsal (e.g., pronouncing the words several times) and spaced exposures (Dempster 1987), such as encountering the words on different occasions in reading materials and conversations.

Several sophisticated systems have been developed in order to support context awareness in learning (Ogata et al. 2008; Chen et al. 2009; Petersen et al. 2009). Context is important to vocabulary learning because students can use it for forming stronger associations between the new word and the objects in the real world (Ogata et al. 2011). In vocabulary learning, context can take many forms. Researchers have used personalized learning systems that tailor-fit the vocabulary content to students’ internal context, i.e., their current level of competence (Yang 2012). Researchers have also built vocabulary applications that have capitalized on external, physical contexts, such as studying in a library or eating in the cafeteria (Scott and Benlamri 2010).

### Systems using the environment as context

Situated cognition argues that knowledge cannot be abstracted from the situation from which it was learned. Learning is always embedded in the activity, context, and culture from which the knowledge was developed (Brown et al. 1989). Learning vocabulary words from dictionary definitions and a few sample sentences is inferior to conversations and meaningful bodies of text. The words that students find useful and words they actually use have better chances of getting acquired. Systems for situated vocabulary learning take advantage of situated cognition by selecting words that are associated with the environment and teaching only the words that are useful. Researchers are taking advantage of near transfer or applying the knowledge learned in a specific situation to an almost similar context (Dunleavy and Dede 2014). In situated vocabulary learning, the words are learned in the context of its use thus facilitating knowledge transfer. Moreover, it encourages the students by illustrating the relevance of the vocabulary words.

Language is always situated in activities that are bound to an environment with its accompanying physical, social, and cultural aspects. In two case studies, Wong and Looi (2010) asked students to take pictures that illustrate English prepositions and Chinese idioms. For 9 weeks, students used mobile phones to take pictures in school and at home. They then annotated the pictures with sentences. These sentences were shared and revised with classmates thereby making the activity collaborative. In their study with 40 students, they have gathered 481 photo-sentence pairs, 124 revisions, and 134 comments. Although the students enjoyed the activity, they observed that there is a wide variability in student participation. Students contributed an average of 12.0 (SD = 25.9) pictures and each offered the revision of 3.1 (SD = 7.3) sentences.

Researchers explain that ubiquitous, context-aware systems are useful for facilitating situated cognition (Brown et al. 1989). To provide location-aware systems, researchers have described wireless positioning techniques and content distribution using the wireless local area network (WLAN) within their campus (Hsieh et al. 2007; Al-Mekhlafi et al. 2009; Epp 2013). Using the campus WLAN, Liu (2009) provided the content for HELLO, an English language learning system. The system detects the location of the user using quick response (QR) codes spread around the school. At each location, students practiced conversations with a virtual learning tutor. In their user testing with 64 students, they report that the students who used the situated language learning approach scored significantly higher (*M* = 89.4, SD = 7.5) compared to those who used printed materials and audio recordings (*M* = 81.3, SD = 9.6). The observed large effect (*d* = 1.0) is attributed to practicing English in real-life situations and encouraging the creativity of the students in handling conversations.

Instead of using WLAN positioning techniques and QR codes, Edge et al. (2011) took advantage of the sub-categories of Foursquare^1^ as the classification of the type of establishment the user is currently in. They then generated the vocabulary words that are frequently associated with that establishment. The users study these vocabulary words via a mobile application called MicroMandarin. For 4 weeks, 23 participants used their system to learn Chinese vocabulary words in establishments in Shanghai and Beijing. Of all the participants, 68 % felt that the detection of their location was “ok” to “great” and 91 % found that the vocabulary content was “ok” to “great.”

Similar to MicroMandarin, Vocabulary Wallpaper (Dearman and Truong 2012) is a microlearning mobile application that takes advantage of idle times that people spend waiting in different locations. Dearman and Truong prototyped the Vocabulary Wallpaper for casual learning of Italian users in three types of establishments within the vicinity of their university. Using GPS or network positioning, Vocabulary Wallpaper determines which of the predefined establishment the user is in. The researchers tested the application with 16 participants using it for four sessions. The results show that the participants can recall an average of 23.3 (SD = 17.1) words and recognize an average of 39.5 (SD = 19.3) words out of all the 75 words. Interestingly, the participants significantly (*p* < 0.05) gained more situated words (*M* = 9.27, SD = 6.44; *M* = 7.33, SD = 5.68) than words that were designed to appear more frequently (*M* = 6.73, SD = 6.17).

Aside from presenting information related to the user’s current environment, the tagged added learning object (TANGO) system uses RFID to tag the objects in the environment to present vocabulary words relevant to an object. They equipped a PDA with an RFID reader which scans the environment. A question is presented to the users on the PDA and they answer by tapping their PDAs to the correct object. They evaluated the usability of TANGO in two user studies. In the first user study with six students (Ogata and Yano 2004), TANGO has a perceived ease of use of 3.3/5 (SD = 1.0) and a perceived usefulness of 4.2/5 (SD = 0.4). In the second user study with 16 students (Ogata et al. 2010), TANGO improved its perceived ease of use at 4.3/5 and perceived usefulness at 4.7/5.

Beaudin et al. (2007) took TANGO to the next level by detecting more user interactions with objects inside a house. Aside from tagging objects with RFID, they used three more sensors: switches for opening and closing cabinets, water flow detectors for the plumbing system, and piezo-triggered accelerometers to detect movements of objects. Overall, they tagged over 100 objects inside the house with 400 Spanish phrases. The system identifies the users through their mobile phones. When they use a particular object (e.g., open a door, sit on a sofa), the system plays the relevant English word and its Spanish translation. If they want to browse previously encountered content, they can access the phrases through their mobile phones. They asked a couple to use the system for 10 weeks. On the average, the phrases where presented 57 times per hour. However, even at this intense interaction, the couple found it acceptable even for extended use. The male participant recalled 158 of the 274 phrases he encountered and he correctly guessed 65 out of the 126 phrases that were not presented to him. The female participant recalled 79 of the 178 phrases presented to her and she correctly guessed 26 of the 92 phrases that were not presented to her.

### Connecting vocabulary and the environment using augmented reality

There are several ideas using AR technology to motivate language learning. For example, Li et al. (2014) made a flash card interaction for learning English. Our idea is to use AR for situated vocabulary learning. The most important feature of situated vocabulary learning is the presentation of useful vocabulary words relevant to the current environment. Based on the ARCS model (Keller 1987), relevance is one of the four factors to consider in creating motivating instructional materials. ARCS stands for attention, relevance, confidence, and satisfaction which are the factors contributing to motivation in using learning materials. Among Keller’s suggestions is relating new information to something the student is familiar with. In our case, we relate the vocabulary words with a familiar environment.

Existing applications can already deliver the relevant and useful information. However, the visualization of information remains on the mobile phone screen without showing the relationship with the real environment. The users are expected to find the relationship of the vocabulary to their surroundings. This relationship is not always obvious. Using AR, we improved the presentation method by annotating real objects with sound, text, images, and animations that are 3D-registered onto the environment. This kind of visualization is beneficial to situated vocabulary learning because it explicitly illustrates the relationship of the vocabulary with the objects found in the current environment.

## Multimedia learning applied to augmented reality

In multimedia learning theory, multimedia refers to pictures and words (both written and spoken). It has three assumptions, namely, dual-channels, limited capacity, and active processing. First, humans have two separate channels for perceiving visual and auditory information. Second, individuals can only attend to a limited amount of information at any given time. Lastly, learning only takes place if the learner actively processes incoming information by connecting it to prior knowledge. Multimedia learning identifies five processes (Mayer 2009; Mayer 2005) in learning:

1. Selecting words
2. Selecting images
3. Organizing selected words
4. Organizing selected images
5. Integrating information with prior knowledge

### Implications of multimedia learning on augmented reality

Situated vocabulary learning leverages on the prior knowledge of places. Visualizing the information in context-rich environments using AR can aid students in creating meaningful associations between the content and the real environment. This promotes having a more elaborated knowledge and having more memory retrieval cues. Situated multimedia aids in the cognitive process of integrating incoming information with prior knowledge. This is consistent with the findings of Fujimoto et al. (2013, 2012).

However, AR is also prone to presenting too much information and too much context from the environment leading to cluttered displays (Peterson et al. 2009; Grasset et al. 2012). This problem arises from the fact that the environment cannot be controlled by the author of the content, whereas all other types of multimedia (books, computer-based media, virtual environments, etc.) give authors full control of the content. For example, they can make an illustration as abstract or as contextualized as they like by removing or adding some details. In the case of AR, the environment is a given and authors of AR learning contents must make use of the environment creatively.

Cluttered displays hamper the cognitive processes of selecting and organizing. As such, in order to benefit from AR visualization, we need to make sure that we design against visual clutter for our AR application. We can confirm if we are successful or not with the design by conducting usability evaluations (Gabbard and Swan 2008). To conduct usability evaluations, we can use a general system usability questionnaire like the system usability scale, or SUS (Lewis and Sauro 2009). Another useful tool is the Handheld Augmented Reality Usability Scale, or HARUS^2^ (Santos et al. 2014c; Santos et al. 2015a), which has a comprehensibility component which measures the ease of understanding an AR visualization.

### Multimedia learning studies in vocabulary learning

Given that individuals have a limited capacity for attending to information, Lin and Yu (2012) investigated the cognitive load induced by different types of media presentations on a mobile phone. In their study with 32 eighth graders, they investigated the use of four multimedia modes, namely, text, text with audio, text with picture, and text with audio and picture. They discovered that the multimedia mode does not have a significant effect on vocabulary gain and retention. However, the learners rated the combined text-audio-picture as the mode that induced the least cognitive load.

Lin and Wu (2013) investigated the use of these four multimedia modes in a succeeding study with 423 junior high school students. They did not find any significant differences in vocabulary recognition nor in any interaction between multimedia modes and learning style preferences of the students. However, the participants who used text with audio and picture performed best in listening tests followed by the text with sound group. This result confirms the intuition that audio annotations contribute to the construction of phonological knowledge of words and the application of this knowledge in listening to sentences. More importantly, they reported that the learning effects of the audio were maintained for 2 weeks with minimal attrition. Based on these works, we implemented features in our AR system that allow users to access text, audio, and pictures during the learning scenario.

In a separate study with 121 senior high school students, Lin and Hsiao (2011) studied the effects of the use of still images against simple animations in vocabulary learning. Their results showed that the animation group performed significantly better in learning Chinese and English vocabulary words compared with the image group. They recommended the use of animations to illustrate dynamic words and processes. Thus, to facilitate better understanding of vocabulary in our handheld AR system, we included a feature wherein sprite sheet animations can be used. We found this feature to be a simple solution to illustrate verbs in our learning scenario.

## Practical considerations in applying augmented reality

Aside from providing evidence of some benefits in the learning process, AR must also adhere to some practical considerations in order to adopt them in actual use. Cuendet et al. (2013) shares five design principles for adopting AR for classroom use. The five design principles are integrating AR to other class activities, empowering the teacher, providing the teacher awareness of the state of students, flexibility to adapt the activities to evolving scenarios, and minimizing functionalities to what is required at a given time.

Based on a survey with teachers and students in Malaysia, Sumadio and Rambli (2010) observed that although most of them experienced AR for the first time, they perceived that the demonstrations presented to them are useful for educational practice. The prototype they showed was an AR learning experience for physics experimentation on heat absorption. Teachers and students expressed that bringing AR to educational use would make the learning process more enjoyable. The other perceived benefits are having a better visualization and being able to simulate an experiment before the actual one. From this example, the participants suggested that it is better to improve the realism of the virtual objects and expand the prototype to cover other experiments that are within the Malaysian physics curriculum.

Based on interviews with teachers in the Philippines (Santos et al. 2013; Santos et al. 2015b), AR is perceived to be useful because it offers learning by experiencing some activity that cannot be done now in the classroom. In these works, researchers are developing an AR X-ray system for looking inside objects and inspecting occluded structures. Currently, even younger learners use desktop computers, smartphones, and gaming consoles in their daily life. Although more conventional mediums of instruction will always remain relevant, the teachers would like to take advantage of various technological interventions to connect with their students. Currently, the teachers are interested in using AR to motivate class participation and to hold the attention of students. This sentiment echos the “empowerment” design principle of Cuendet et al. (2013) which states that the teacher should remain the central point of class interaction.

However, the teachers also expressed their concerns about the use of AR technology. In order to adopt AR technology for the classroom in the next few years, engineers should consider the cost of the technology, usability, and time constraints, including the time to set up and covering the required materials for class. This feedback is related to the “minimalism” design principle of Cuendet et al. (2013) which dictates that the functionalities engineers should provide must be limited to what is required. More functionalities than required would make AR more difficult to use.

## Design goals

To summarize what we discussed so far, we list the following design goals based on multimedia learning, past works on situated vocabulary learning, and some practical considerations for future adoption to educational settings:

1. Minimize visual clutter on the display
2. Support cognitive processes of selecting, organizing, and integrating information
3. Allow interactions with the environment and objects in the environment
4. Present multimodal information, namely, texts, images, and sounds
5. Use animations when appropriate
6. Apply cheap and accessible technology
7. Make the contents easy to create
8. Limit the interactions

## System design and implementation

We created a handheld AR system that can display any combination of multimedia including image, animation, sound, and text on a real environment. We then created two AR applications for learning Filipino and German words in a real environment. We accomplished this by simply filling the handheld AR system with content for the situated vocabulary learning of Filipino and German words.

### Handheld augmented reality system

**Fig. 1:**
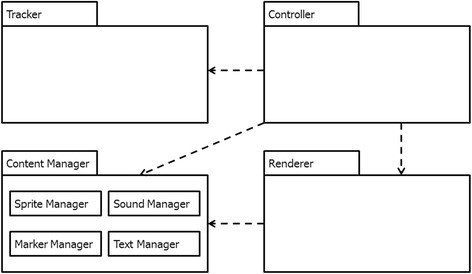
Package diagram of our handheld augmented reality system (Santos et al. 2014b)

**Fig. 2:**
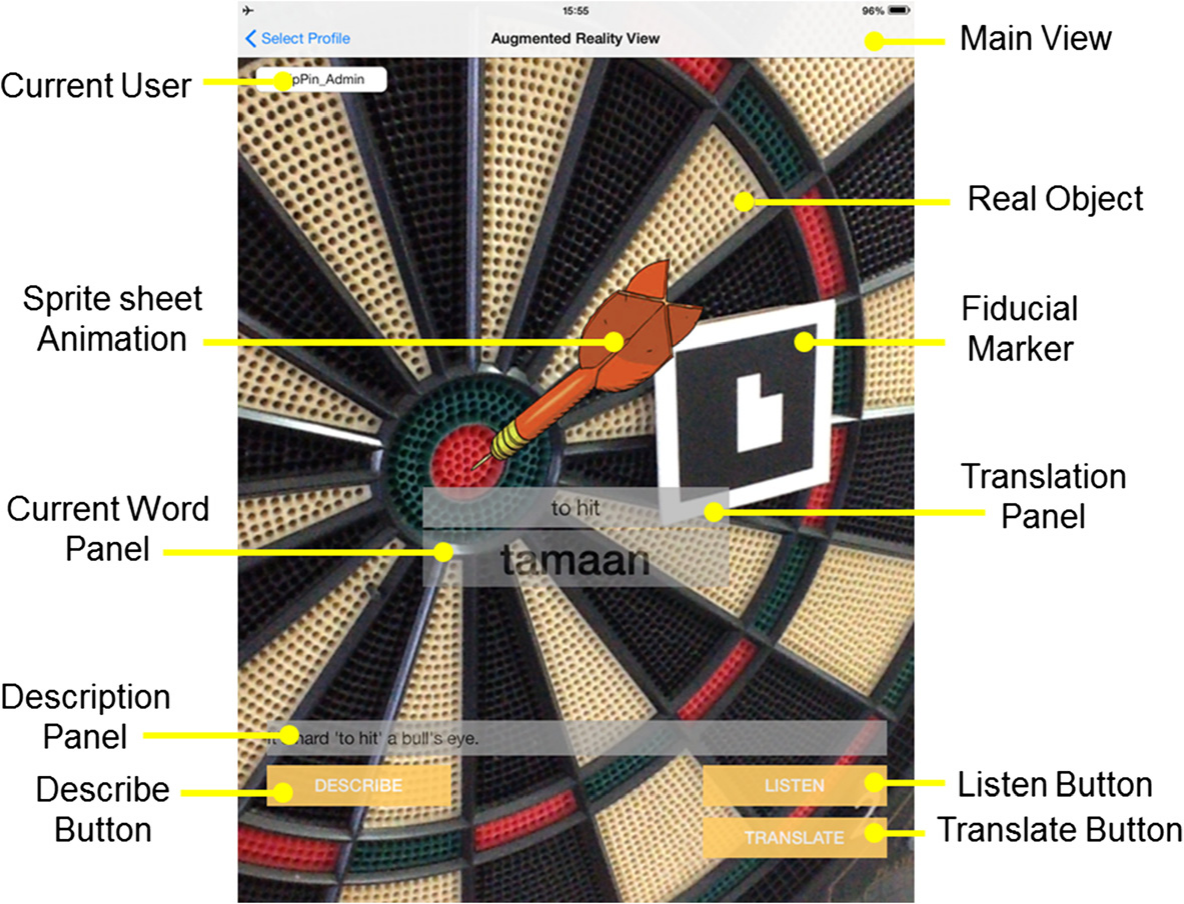
Sample interface for situated vocabulary learning

Figure 1 shows the package diagram of our system and Fig. 2 shows the sample interface enabled by our system. The main part of the system is the Controller, which has access to learning contents, sensors (cameras), and user inputs. The Controller receives the marker ID and camera view matrix from the Tracker and uses these information to specify the behavior of the on-screen display. The Tracker was built using ARToolKit and the Renderer was built on OpenGL ES 2.04.

We used ARToolKit (Kato and Billinghurst 1999) to measure the camera pose with respect to the target object. Fiducial markers in the video feed were located using the ARToolKit, which also outputs the marker’s ID and the matrix representing the current view of the camera. The image was transformed to the correct view using the matrix, and then it was rendered accordingly using OpenGL ES 2.04.

**Fig. 3:**
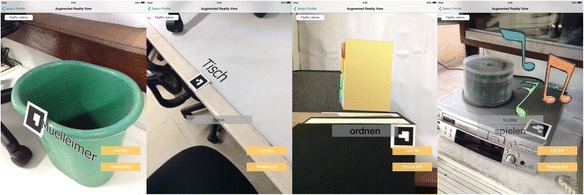
Displaying nouns using labels and displaying verbs as animations on real objects

The AR system runs entirely on iPad tablets. For our experiments, we used the iPad 2 (dual-core A5, 512-MB DDR2 RAM, 32 GB, 601 g, 9.7 in display, 1024 × 768 at 132 ppi) and the iPad mini (64-bit A7, 512-MB DDR2 RAM, 16 GB, 331 g, 7.9 in display, 1024 × 768 at 163 ppi). The system works with fiducial markers (Fig. 3) to determine the target object and the viewing angle of the tablet’s back camera. We used the back camera set to 640 × 480 pixels at 30 fps to sense the marker and to provide a video feed. After identifying the marker, the system loads the corresponding audio, text, and image. Audio and text can be accessed using buttons (LISTEN, TRANSLATE, DESCRIBE). The images can either be still images or sprite sheet animations (Figs. 1 and 3). The images are transformed depending on the camera view and are inserted in the video feed to suggest 3D registration, that is, to give an impression that the graphics co-exist with the real objects.

### Situated vocabulary learning content

We used the AR system to construct two situated vocabulary learning systems: one for 30 Filipino words and the other for 10 German words. We based the design of the content from previous works (Lin and Hsiao 2011; Lin and Yu 2012; Lin and Wu 2013) by using a combination of text, audio, images, and animations as content. The text data are the vocabulary, its translation in English, and the description of the scene (only for the Filipino version). The audio data is the proper pronunciation of the vocabulary as spoken by a native speaker. The image data are text labels, images, or animations, as shown in Fig. 3.

## User studies

**Fig. 4:**
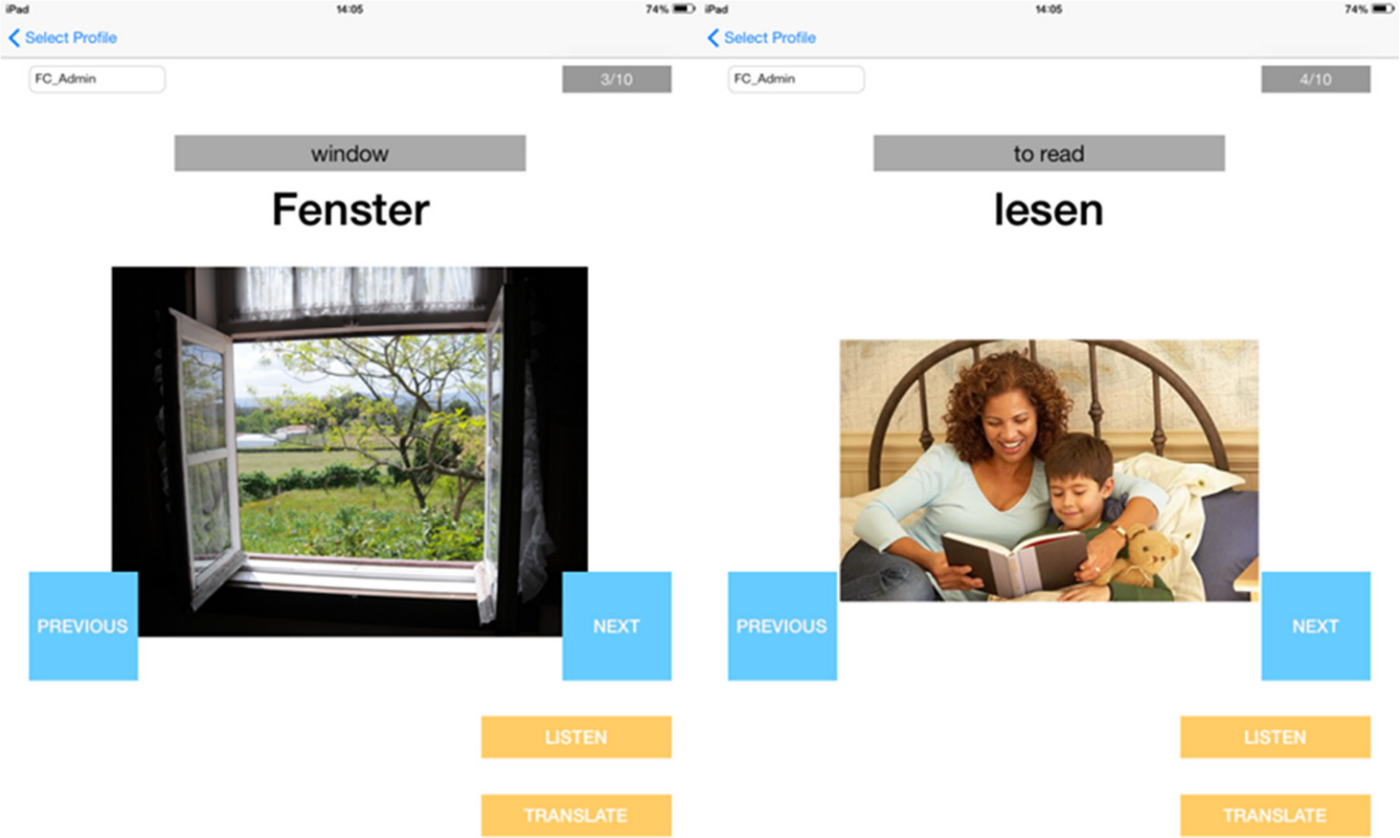
Non-AR version of the AR applications

We explored the strengths of our AR applications for situated vocabulary learning over its non-AR counterpart (Fig. 4) in two preliminary experiments. In particular, we are interested in the effects of AR on memorization and student motivation. Through these experiments, we aim to evaluate the use of AR for viewing vocabulary content that is situated in the real environment. We compared the AR applications to a non-AR version which is a tablet application that mimics flash card interaction. Our comparison does not employ any kind of special instructional design, such as game mechanics and collaboration. As summarized in Table 1, users simply point the tablet PC to objects found in their environment when using our AR application. On the other hand, the flash card application allows the user to flip through contents by pressing either next or previous.

**Table 1:** Summary of comparison of two interfaces for vocabulary learning

	AR application	Non-AR application
Interaction	Users find an object with a marker. They then point the tablet PC to the marker to reveal the content.	Users press “next” or “previous” to switch between contents.
Inherent feature	Users can see the markers in their environment even when they are not studying.	Users can quickly go through all the material because they are arranged in a series.
Visual display	Texts, images, sounds, and animations are displayed in the real environment.	Illustrations are shown on a white background.
Place and time	Users can only use it inside their laboratory at any given time.	Users can only use it inside their laboratory at any given time.

We considered inherent features of the interaction as part of the treatment. Thus, we made no attempts to control them. For example, one advantage of an AR learning system is that the students see the real objects in their surroundings even when they are not studying. We imagine this feature to trigger unintended rehearsal of the vocabulary, thereby improving memorization. This unintended rehearsal is part of AR learning, thus, we did not control this aspect. We did not forbid the students in the AR treatment from visiting the study place when they were not studying.

Another inherent feature is that students tend to cover all the vocabulary words several times in one study session when flash cards are used. The flash cards are sequentially arranged and students try to go through all the content two to four times in one sitting. Even if this is the case, interventions were not made because it is an inherent feature of the use of flash cards. Moreover, advising the students who use the AR application to view all the content several times will interrupt their natural learning style.

For our experiments, we controlled both location and time constraints. All of our students were only allowed to use the applications inside their respective laboratories. However, the applications are available to them at any time they want to study on that day. Given these features, we had seven hypotheses which we tested for significance in the 0.05 level via Student’s *t* test and analysis of covariance (ANCOVA). The hypotheses are as follows:

1. Students will perform worse on a delayed post-test with non-AR compared with the immediate post-test.
2. Students will perform worse on a delayed post-test with AR compared with the immediate post-test.
3. Students will perform better in an immediate post-test with non-AR.
4. Students will perform better in a delayed post-test with AR.
5. Students will rate AR as a more motivating instructional material.
6. Students will maintain their attention better with AR.
7. Students will find the contents presented through AR to be more relevant to them.
8. Students will feel more confident with non-AR.
9. Students will feel more satisfied with AR.

### User testing 1: learning 30 Filipino words in 5 days

We adapted a between-groups approach with 31 participants (26 male, 5 female, aged 23–42, information science graduate students) to test our application for studying Filipino words. The first languages of the participants are Japanese (13), Chinese (5), Portuguese (3), German, English, Turkish, Bosnian, Indonesian, Finnish, Arabic, Spanish, Nepali, and Wolof. In our experiments, we divided the people into the treatment groups with consideration to the distribution balance of their first languages.

**Fig. 5:**
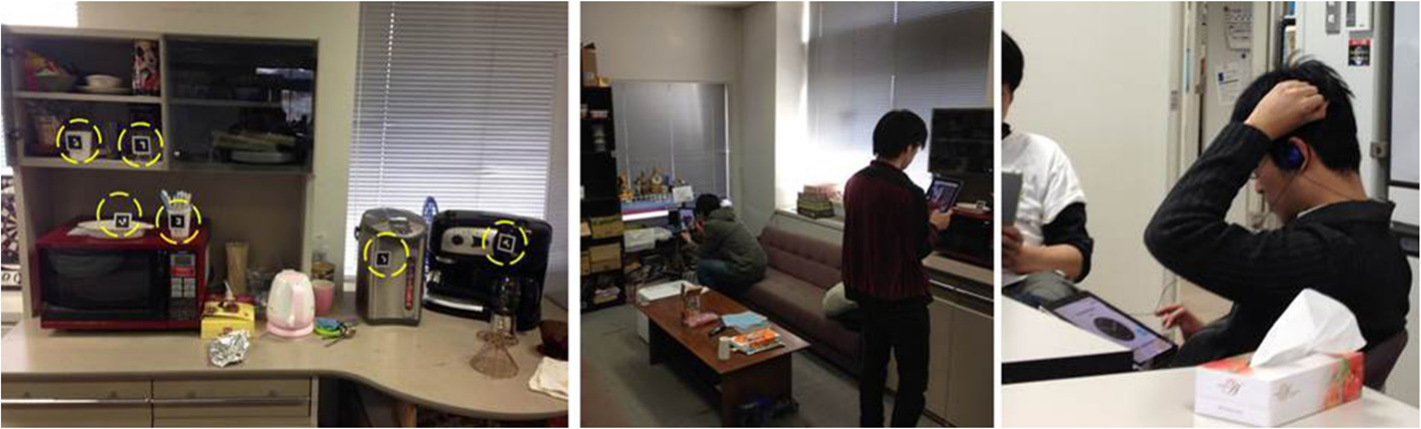
Refreshment area with markers (*left*), learners using situated vocabulary learning (*middle*), learners using non-AR vocabulary learning (*right*) (Santos et al. 2014b)

Eighteen participants were recruited from one laboratory. We set up our system inside their laboratory (Fig. 5) so that they can learn words related to their refreshment area. All of them have experienced using an AR application before, thus, AR is not a novel technology for them. Thirteen participants from three laboratories were asked to use the non-AR version. Similar to the AR group, the non-AR group had used AR before and they are familiar with other novel interfaces. We distributed tablet computers to them with the flash card application installed.

The participants used the assigned application for a recommended duration of 10–15 minutes per day for 5 days. The AR version was used inside a refreshment area with a maximum of four people using the application at the same time (Fig. 5). On the other hand, the learners used the non-AR version whenever they are in their laboratory office.

For our comparative analysis, we evaluated the participants’ learning outcomes and the usability of the application. On the fifth day, the participants answered the SUS to measure the perceived usability of the applications. They then immediately took a post-test. After 12–14 days, they took a delayed post-test. The immediate post-test (27 items) and delayed post-test (24 items) consist of questions on recognizing the word in a multiple choice question, recalling the translation of the word, and guessing which word fits in different contexts.

For further analysis of the usability of our AR application, we asked the participants in the AR group to answer the HARUS which measures general system usability, ease of handling the AR application, and ease of understanding the portrayed information. Lastly, both AR and non-AR applications logged time-stamped button pushes, words studied, and tablet acceleration and orientation based on the built-in sensors. We did not notice any burden on the application due to the system logging even after extended use.

### User testing 2: learning 10 German words

We adapted a within-subjects design with 14 participants (8 male, 6 female, aged 17–20, Filipino undergraduate students) to test the application for learning 20 German words (10 for AR and 10 for non-AR). Each participant used the AR and non-AR versions for a maximum of 8 minutes. Seven used the AR version first, whereas the other seven used the non-AR version first to balance any effect of the ordering of the treatment. For the AR version, the learners viewed the content on a small area around a laboratory technician’s desk. The markers were placed near each other in a small area to minimize the time spent on transferring from one object to another. This was important because we wanted to observe the study time of the students. For the non-AR version, they used the application while sitting inside the same room.

**Fig. 6:**
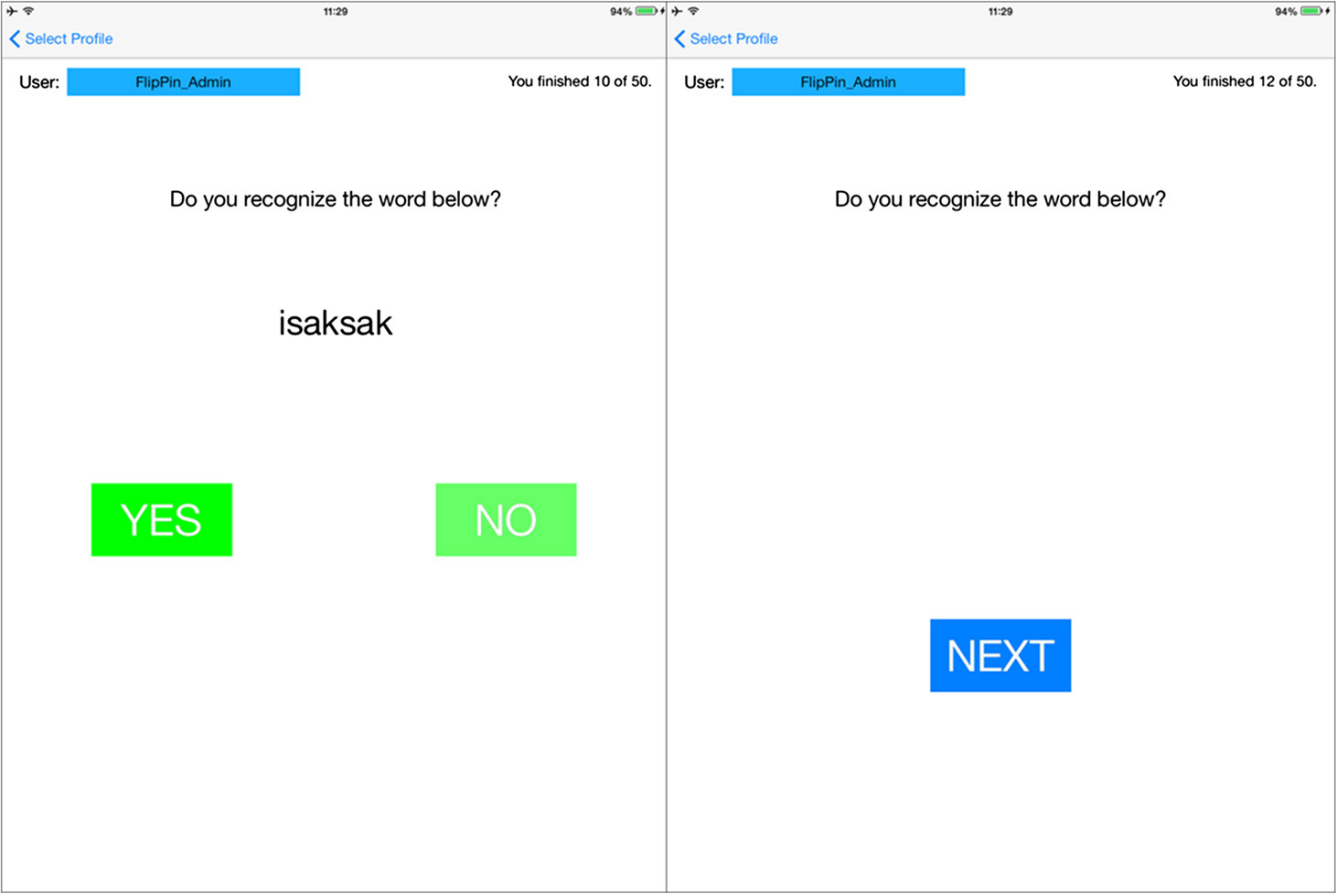
Screen capture of the recognition game

The students are then asked to answer 10 multiple choice questions that test their skill to recognize a word using a recognition game (Fig. 6). Aside from logging the answer, we also logged the time it took for the learner to answer the question. After taking the quiz, the participants also answered a subset of the Instructional Materials Motivational Survey, or IMMS. We picked 30 questions that are applicable to our system out of the 36 questions listed in the work of Huang et al. (2006). IMMS models the extent of motivation one gets from an instructional material by using the ARCS model. This model had been previously applied to AR instructional materials by Di Serio et al. (2013).

## Results and discussion

Our experiments involved a small sample size, thus, the results should be interpreted with caution. These experiments should be replicated with a bigger sample size. Nevertheless, these results can guide future design of AR applications and experiments in situated vocabulary learning with AR. In our experiments, we observed significant decrease in immediate to delayed post-test scores with non-AR but not for AR, suggesting that students who learned via AR retained more vocabulary. No significant differences were observed in learning outcomes between using AR and non-AR applications for vocabulary learning. However, students report better attention and satisfaction in using our system. In summary, we found evidence that supports hypotheses 1, 3, 6, and 9 but not 2, 4, 5, 7, and 8.

### Marginally significant differences in usability and learnability

We computed the SUS score and its factors from the participant responses in experiment 1. The AR application scored 74 on the SUS, whereas the non-AR application scored 80, as shown in Table 2. According to Sauro’s classification (2011), both interfaces were above average (SUS score >68). Thus, both interfaces are good. Moreover, the results in Table 3 show that our participants did not have difficulty in learning how to operate these new interfaces.

**Table 2:** Summary of system usability scale scores

	Application	Number	Mean	SD	*T* value	*p* value
SUS score	AR	18	74	12	1.64	0.055
	Non-AR	13	80	6

**Table 3:** Summary of SUS factor scores

Factor	Application	Number	Mean	SD	*T* value	*p* value
Usability	AR	18	70	14	1.50	0.073
	Non-AR	13	76	7		
Learnability	AR	18	90	13	1.53	0.068
	Non-AR	13	96	5		

We found a marginally significant difference between the two interfaces with a moderate effect size (*d* = 0.63). Despite the differences in usability, using these applications for comparison is reasonable because both represented our best effort and had above average usability. We achieved a good usability score because we applied insights from previous research in multimedia learning. Furthermore, our current interface features were minimal, and the task was simple.

### More pronounced decrease in post-test scores for non-AR

Table 4 is a summary of the results comparing the immediate and delayed post-test scores in experiment 1. For the AR group, six people were not able to take the delayed post-test because they were inaccessible. (They were out-of-town at the time and did not check their emails 12 to 14 days after the study phase.) Both AR and non-AR groups decreased from immediate to delayed post-test scores. The difference for the non-AR group is significant with a large effect (*d* = 0.84), whereas the differences for AR is marginally significant, with a small effect size (*d* = 0.14). Thus, we found evidence supporting hypothesis 1 but not hypothesis 2.

**Table 4:** Comparing immediate and delayed post-tests

Application	Post-test	Number	Mean (%)	SD (%)	*T* value	*p* value
AR	Immediate	18	71	20	1.46	0.058
	Delayed	12	68	23		
Non-AR	Immediate	13	86	20	3.42	0.001
	Delayed	13	70	18		

These results are consistent with the work of Fujimoto et al. (2013, 2012) which reports that information associated with a place is better remembered. In our case, vocabulary that is associated with a place is better remembered than those that were abstracted (non-AR). However, we believe that an experiment with high sample sizes is necessary in order to better support this claim and to better understand how familiar places contribute to the integration process of multimedia learning.

### Significantly higher scores with non-AR for immediate post-test but not for the delayed post-test

Table 5 compares the immediate and delayed post-tests in experiment 1 for AR and non-AR. In the immediate post-test, the non-AR group scored significantly higher with a moderate effect (*d* = 0.75) compared with the AR group, thus supporting hypothesis 3. The breakdown in Table 6 shows that the AR group scored lower than the non-AR group in all types of questions. This result is indicative of an overall inferior mastery of content rather than a weakness in a particular question type.

**Table 5:** Comparing scores with AR and non-AR

Post-test	Application	Number	Mean (%)	SD (%)	*T* value	*p* value
Immediate	AR	18	71	20	2.14	0.020
	Non-AR	13	86	20		
Delayed	AR	12	68	23	0.31	0.380
	Non-AR	13	70	18

**Table 6:** Immediate post-test scores for each question type

Question type	Application	Number	Mean (%)	SD (%)	*T* value	*p* value
With illustrations	AR	18	87	12	0.99	0.163
	Non-AR	13	92	20		
Recognizing Filipino with choices	AR	18	80	15	2.54	0.008
	Non-AR	13	94	15		
Recognizing Filipino without choices	AR	18	64	30	1.95	0.031
	Non-AR	13	83	24		
Translating from English to Filipino	AR	18	55	31	2.54	0.008
	Non-AR	13	81	23		
Transfer word usage with choices	AR	18	75	19	2.40	0.012
	Non-AR	13	91	16		

In most practical cases, people do not usually apply their learning immediately after studying. Rather, they would use their knowledge after a few days, either for a test or to apply it to a new lesson. As such, the delayed post-test is a more important point of comparison for learning than the immediate post-test. After 12–14 days, the significant difference in learning disappeared (Table 7). This is consistent with the results of Lin and Yu (2012) who reported that various multimedia modes did not have significant differences. However, the students did report differences in cognitive load. In experiment 1, the participants are graduate students who may not be sensitive to differences in cognitive load induced by an interface. For experiment 2, we asked a younger group of students to test our interface because they may be more affected by differences in cognitive load induced by interfaces.

**Table 7:** Delayed post-test scores for each question type

Question type	Application	Number	Mean (%)	SD (%)	*T* value	*p* value
With illustrations	AR	12	71	27	0.26	0.400
	Non-AR	13	73	16		
Recognizing Filipino with choices	AR	12	67	23	0.70	0.247
	Non-AR	13	72	13		
Recognizing Filipino without choices	AR	12	69	30	0.09	0.463
	Non-AR	13	71	27		
Translating from English to Filipino	AR	12	65	28	0.10	0.462
	Non-AR	13	64	33		
Transfer word usage with choices	AR	12	64	25	0.87	0.196
	Non-AR	13	71	19		

### No significant differences in immediate post-test scores after considering usability as covariant in ANCOVA

Assuming that implementation quality was a factor affecting the learning of the students, we could do fairer comparisons of post-test scores if both AR and non-AR applications have almost the same SUS score. However, we observed a difference of six SUS points between the AR and non-AR applications. We conducted ANCOVA to take into account this difference in usability.

We can conduct ANCOVA because the difference in SUS score was not significant. We also checked the homogeneity of variances using Levene’s test. The results of Levene’s test showed that there are no significant differences (*p* > 0.05) in variances. The ANCOVA results in Table 8 are almost similar to the ANOVA results in Table 5. Marginally significant differences were observed in the test scores of AR and non-AR groups for the immediate post-tests. However, there is almost no difference in the delayed post-tests.

**Table 8:** Analysis of covariance of post-test scores with system usability scale score as covariant

Post-test	Application	Number	Mean (%)	SD (%)	Adjusted mean (%)	*F* value	*p* value
Immediate	AR	18	71	20	72	3.02	0.09
	Non-AR	13	86	20	85		
Delayed	AR	12	68	23	69	0.00	1.00
	Non-AR	13	70	18	69		

### Differences in usage of AR and non-AR applications

To gain further insight regarding the differences between studying with AR and non-AR applications, we calculated the total amount of time the application is open and the total number of button pushes for LISTEN, TRANSLATE, and DESCRIBE buttons. We found that the non-AR application was used significantly longer compared to the AR application (Table 9), a finding we already expected after observing the participants’ study on the first day and on the fifth day.

**Table 9:** Duration of application use (in minutes)

	Application	Number	Mean	SD	*T* value	*p* value
Usage	AR	18	29.7	10.7	2.88	0.004
	Non-AR	13	55.8	36.5		

In order to study with the non-AR application, the students had to keep the application open for the entire study period. However, when studying with AR, the students could put the application down and rehearse the words by going through each object in the room and calling out the vocabulary. In this case, using the application becomes unnecessary because the room itself represents the learning material. We think this connection with digital content and the real environment is one important feature of AR that could be exploited in ubiquitous learning.

We also found some differences in the amount of buttons pushed in the AR application compared with the non-AR counterpart. All the three buttons (LISTEN, TRANSLATE, DESCRIBE) where used more in general, with the TRANSLATE button being pushed significantly more. This could mean that AR may be more motivating for students, especially for maintaining attention as Di Serio et al. reported (2013). In another study, Ibanez et al. (2014) reported AR’s influence on learners’ flow state, specifically on concentration, distorted sense of time, sense of control, clearer direct feedback, and autotelic experience. As such, for experiment 2, we applied the IMMS similar to Di Serio et al. (2013) to observe motivation. For experiment 2, we removed the DESCRIBE button because students did not use it so much and we did not see any significant differences in its use (Table 10).

**Table 10:** Total buttons pushed

Button	Application	Number	Mean	SD	*T* value	*p* value
Listen	AR	18	408	364	1.01	0.160
	Non-AR	13	262	168		
Translate	AR	18	40	23	2.32	0.015
	Non-AR	13	16	23		
Describe	AR	18	69	70	0.35	0.365
	Non-AR	13	58	88		

### No significant differences in recognition test, but significantly better attention and satisfaction with AR

There was no significant difference between the recognition test between using AR (*M* = 94 %, SD = 8 %) and using non-AR (*M* = 95 %, SD = 8 %) for vocabulary learning. On the average, the non-AR group answered our multiple questions faster (*M* = 2.28 s, SD = 0.92 s) than the AR group (*M* = 2.60 s, SD = 1.03 s) for each question. However, this difference was not significant.

Experiment 2 focuses on evaluating motivation by using the ARCS model. Although two interfaces can arrive at the same learning outcome, performance in tests should not be the only measure of success in creating interfaces. User experience is another important consideration. As such, we also evaluated the interfaces in terms of its ability to motivate students to learn.

Overall, we only observed a marginally significant difference between the IMMS rating of AR and non-AR vocabulary learning (Table 11). However, looking at the factors of the IMMS (Table 12), significant differences were observed in the attention and satisfaction factors. The students report that the AR application catches and holds their attention more than the non-AR application. This is consistent with the observations of Di Serio et al. (2013). Moreover, they report higher satisfaction with their learning experience. The learners were slightly more confident to use non-AR probably because it is a more familiar interface. This finding is opposite of that of Di Serio et al. (2013). The learners rated AR to be higher in relevance by five points, which is attributed to the implicit connection between learning contents and real environment. However, no statistical significance was observed for the relevance and confidence factors.

**Table 11:** Summary of the Instructional Materials Motivational Survey scores

	Application	Number	Mean	SD	*T* value	*p* value
Motivation score	AR	14	76	12	1.34	0.096
	Non-AR	14	71	11

**Table 12:** Factors of the Instructional Materials Motivational Survey score

Factors	Application	Number	Mean	SD	*T* value	*p* value
Attention	AR	14	75	14	1.84	0.038
	Non-AR	14	65	14		
Relevance	AR	14	74	14	0.97	0.172
	Non-AR	14	69	13		
Confidence	AR	14	80	12	0.74	0.232
	Non-AR	14	83	8		
Satisfaction	AR	14	77	16	1.71	0.049
	Non-AR	14	66	18		

### Usability, manipulability, and comprehensibility of our AR application for situated vocabulary learning

Aside from the system usability scale, we used HARUS (Santos et al. 2014c; Santos et al. 2015a) to measure the system usability of our system. HARUS is specifically designed for handheld AR. It has two factors relevant to AR, namely, manipulability and comprehensibility. Manipulability corresponds to the ease of handling the device when doing certain tasks. Usability questionnaires for software and mobile phones do not usually cover manipulability because software tends to be stationary and mobile phones tend to be held with a fixed posture. AR, on the other hand, requires the user to move around while pointing their handheld devices at various angles. This can be difficult sometimes due to unstable tracking of the natural environment, among other reasons. The second factor of HARUS is comprehensibility which is the ease of understanding the presented information. Although comprehensibility is common to all types of software, HARUS is designed for users to respond to AR-specific issues, such as the alignment of virtual contents and real environments, visual clutter, and depth perception.

Table 13 summarizes the HARUS score and its factors for the AR application. Our current prototype scored 61 (out of 100) in terms of overall usability, with a score of 63 on manipulability and 59 on comprehensibility. Compared to the usability score of 74, we think that we got a lower usability score from HARUS because it is more sensitive to AR applications. This current score can be used as a reference for the next iteration of our application. It could also be used as a benchmark for other AR applications for situated vocabulary learning. Through the use of HARUS, we may be able to compare handheld AR systems more accurately. However, its results should be interpreted with caution because HARUS is a relatively new questionnaire with some evidence of validity and reliability.

**Table 13:** Summary of HARUS scores and its factors

	HARUS	Manipulability	Comprehensibility
AR	61	63	59

One of the straightforward ways to improve our system is to use lighter devices. Some students reported that the iPad 2 is too heavy for our purpose and it requires the use of two hands. Another way to improve the manipulability of our system is to use some ergonomically designed handle for tablets, such as the work of Veas and Kruijff (2008).

We think that applying markerless tracking, such as point-cloud-based tracking using the PointCloud SDK,^3^ would decrease comprehensibility if we cannot detect good enough features to track the environment. Moreover, such feature registration process would be difficult to create if the content authors are teachers. For our current application, simply printing markers and placing them in the environment is an easier and more stable way of tracking the environment. However, we expect both markerless tracking technology and tablet computing power to improve significantly in the next few years. At that time, switching to markerless tracking would be practical.

## Conclusions

Augmented reality is useful for presenting situated multimedia in ubiquitous learning. In our work, we discussed our experience in developing and evaluating an AR application for learning experiences based on an authentic environment. As part of our development process, we drew design goals from multimedia learning theory, past systems for vocabulary learning, and needs of teachers. We then created a handheld AR system for displaying situated multimedia (text, image, sound, and animation). As a use case of the AR system, we filled the system with Filipino and German vocabulary contents, thereby creating two AR applications for situated vocabulary learning.

We evaluated the AR applications by combining methods in human-computer interaction, usability engineering, and education technology. We observed differences in immediate post-tests wherein students who used the non-AR application performed better than those who used AR. This effect is only temporary as both AR and non-AR users have almost equal scores in the delayed post-tests. We observed a larger difference between immediate post-test and delayed post-test for the students who used the non-AR application. This suggests that using AR resulted in better retention. This result needs to be explored further because our evaluation involved a small sample size only.

Aside from differences in post-tests, the potential of AR lies in the difference in the learning experience, more specifically, reducing cognitive load, improving attention, and increasing satisfaction. Although preliminary, our experiments suggest that AR as multimedia may lead to better attention and satisfaction.

For future work, experiments with bigger sample size must be used to explore deeper into how students can learn better with AR. Moreover, aside from cross-sectional studies comparing AR with a more traditional interface, longitudinal studies are necessary to explore the evolution of students’ knowledge and skills over time.

## Footnotes

1
https://foursquare.com/

2
http://harusability.com/

3
http://developer.pointcloud.io/
